# Complete Genome Sequence of *Aurantimicrobium* sp. Strain INA4, Isolated from an Oligotrophic Lake in Japan

**DOI:** 10.1128/mra.01247-22

**Published:** 2023-02-21

**Authors:** Yusuke Ogata, Keiji Watanabe, Shusuke Takemine, Kaoru Kaida, Maki Tanokura, Wataru Suda

**Affiliations:** a RIKEN Center for Integrative Medical Sciences, Yokohama, Kanagawa, Japan; b Center for Environmental Science in Saitama, Kazo, Saitama, Japan; University of Delaware College of Engineering

## Abstract

The globally distributed freshwater bacterioplankton of the genus *Aurantimicrobium* belong to the tribe Luna2. Here, we report the complete genome sequence of *Aurantimicrobium* sp. strain INA4, which was isolated from an oligotrophic lake surface water in Japan.

## ANNOUNCEMENT

The bacterioplankton of the genus *Aurantimicrobium*, which belongs to the Luna2 tribe, have worldwide distribution in freshwater environments (1, 2). To date, only two species (Aurantimicrobium minutum and Aurantimicrobium photophilum) have been described (3, 4). In comparison with its ubiquity, the genomic information of *Aurantimicrobium* is very limited. Therefore, accessing the genomic information for *Aurantimicrobium* bacteria is important for the estimation of physiochemical properties and ecological insights into the genus.

Here, we report the complete genome sequence of an *Aurantimicrobium* sp. strain, INA4 (JCM 18065), which was isolated from an oligotrophic lake surface water sample collected from Lake Inawashiro, Inawashiro-machi yama-gun, Fukushima, Japan (37°29′53.6″N 140°08′45.3″E), on 20 June 2010. The sample was filtered through a disposable syringe equipped with a 0.7-μm particle-retention glass-fiber filter (Puradisc 25 GF/F disposable filter device; Whatman, Springfield Mill, UK). After filtration, the filtrate was spread onto a modified Reasoner’s 2A (MR2A) agar plate and incubated at 25°C for 5 days (5). A single bacterial colony was picked, inoculated into sterilized MR2A liquid medium (pH 7.2), and incubated at 25°C for 2 days with reciprocal shaking (120 rpm), and the pure strain cell suspension was preserved at −80°C as the stock in MR2A broth supplemented with 20% (wt/vol) glycerol. Cells from the glycerol stock were inoculated and cultured in MR2A liquid medium, harvested by centrifugation, and used for genomic DNA extraction.

Genomic DNA was extracted from strain INA4 using enzymatic lysis and phenol-chloroform-isoamyl alcohol extraction as described previously (6). The library was prepared with the SMRTbell express template prep kit v2.0 (Pacific Biosciences of California, Inc., [PacBio], Menlo Park, CA) with DNA shearing by gTUBE (Covaris, LCC, Woburn, MA) and with a target length of 10 to 15 kb. Whole-genome sequencing was performed using the Sequel II platform (PacBio). The PacBio reads were converted to circular consensus sequencing (CCS) reads using CCS software v.6.2.0 (https://github.com/PacificBiosciences/ccs) and assembled using the assembler Canu v.2.1.1 with specified parameters minReadLength = 2,200 and minOverlapLength = 2,200 (7), and the generated contig was checked for circularization to remap the CCS reads by Minimap2 v. 2.24-r1122 (8). The genomic sequence obtained was annotated and rotated to start at *dnaA* using DFAST (https://dfast.nig.ac.jp) (9). Default parameters were used for all software analysis, unless otherwise specified. The reads obtained and genomic sequence generated are summarized in Table 1.

**TABLE 1:** Summarized data on reads and contig obtained for *Aurantimicrobium* sp. strain INA4

Parameter	Data
Data for quality-checked Sequel reads	
No. of reads	3,542
Total no. of bases	50,195,765
*N*_50_ (bp)	14,069
Total no. of contigs	1
BioProject accession no.	PRJDB14572
BioSample accession no.	SAMD00549753
SRA accession no.	DRR413426
Genome size (bp)	1,581,031
GC content (%)	52.7
GenBank/ENA/DDBJ accession no.	AP027040

Based on annotation results for the strain INA4 genome sequence, a gene predicted to encode polyphosphate kinase (*ppk*), which is associated with the intracellular accumulation of polyphosphate, was identified, along with a gene encoding a putative xanthorhodopsin-like protein associated with a light-driven, proton-pumping rhodopsin. Furthermore, this strain also contained genes encoding putative alanine dehydrogenase and glycine dehydrogenase-like proteins associated with ammonification.

### Data availability.

The chromosome sequence and reads for strain INA4 were deposited in the GenBank/ENA/DDBJ database under the accession no. AP027040, which is linked to the BioProject accession no. PRJDB14572, the BioSample accession no. SAMD00549753, and DDBJ Sequence Read Archive accession no. DRR413426. The details are summarized in Table 1.
